# Integrated Analysis and Visualization of Group Differences in Structural and Functional Brain Connectivity: Applications in Typical Ageing and Schizophrenia

**DOI:** 10.1371/journal.pone.0137484

**Published:** 2015-09-02

**Authors:** Carolyn D. Langen, Tonya White, M. Arfan Ikram, Meike W. Vernooij, Wiro J. Niessen

**Affiliations:** 1 Biomedical Imaging Group Rotterdam, Departments of Radiology & Medical Informatics, Erasmus MC, Rotterdam, The Netherlands; 2 Department of Child and Adolescent Psychiatry, Erasmus Medical Centre, Rotterdam, Netherlands; 3 Imaging Physics, Faculty of Applied Sciences, Delft University of Technology, Delft, The Netherlands; 4 Department of Epidemiology, Erasmus MC, Rotterdam, The Netherlands; 5 Department of Radiology, Erasmus MC, Rotterdam, The Netherlands; Institute of Psychology, Chinese Academy of Sciences, CHINA

## Abstract

Structural and functional brain connectivity are increasingly used to identify and analyze group differences in studies of brain disease. This study presents methods to analyze uni- and bi-modal brain connectivity and evaluate their ability to identify differences. Novel visualizations of significantly different connections comparing multiple metrics are presented. On the global level, “bi-modal comparison plots” show the distribution of uni- and bi-modal group differences and the relationship between structure and function. Differences between brain lobes are visualized using “worm plots”. Group differences in connections are examined with an existing visualization, the “connectogram”. These visualizations were evaluated in two proof-of-concept studies: (1) middle-aged versus elderly subjects; and (2) patients with schizophrenia versus controls. Each included two measures derived from diffusion weighted images and two from functional magnetic resonance images. The structural measures were minimum cost path between two anatomical regions according to the “Statistical Analysis of Minimum cost path based Structural Connectivity” method and the average fractional anisotropy along the fiber. The functional measures were Pearson’s correlation and partial correlation of mean regional time series. The relationship between structure and function was similar in both studies. Uni-modal group differences varied greatly between connectivity types. Group differences were identified in both studies globally, within brain lobes and between regions. In the aging study, minimum cost path was highly effective in identifying group differences on all levels; fractional anisotropy and mean correlation showed smaller differences on the brain lobe and regional levels. In the schizophrenia study, minimum cost path and fractional anisotropy showed differences on the global level and within brain lobes; mean correlation showed small differences on the lobe level. Only fractional anisotropy and mean correlation showed regional differences. The presented visualizations were helpful in comparing and evaluating connectivity measures on multiple levels in both studies.

## Introduction

Brain connectivity is an area of research receiving increasing attention. In the brain, regions communicate through networks of structural and functional links, which is termed connectivity. Connectivity can be studied non-invasively using advanced imaging techniques such as magnetic resonance imaging (MRI). Relationships between regions of interest (ROIs) are extracted from MRI’s and used to define a network. Variations in MRI acquisition and analysis have resulted in several types of connectivity. With ever increasing choices, comparing connectivity types becomes an important first step in addressing a given research question.

*Structural connectivity* refers to the anatomical connectivity of the brain. It can be extracted from diffusion weighted images (DWI). DWI is a type of MRI that estimates the movement of water in each voxel of the image. Water diffuses along white matter tracts. From DWI, pathways travelled by water molecules (and thus the orientation of nerve fibers) can be approximated. These pathways are referred to as streamlines [1]. Measures of microstructure along the length of streamlines, such as fractional anisotropy (FA), which is the degree of diffusion directedness, can be collected and used to define structural connectivity [2]. Structural connectivity measures have also been derived without using streamlines, such as in estimating minimum cost to travel between regions [3,4].

It is believed that structural connections enable communication between regions, resulting in *functional connectivity* [5], characterized by regions having similar neural dynamics. Functional connectivity can be quantified using functional MRI (fMRI). Several methods of defining functional connectivity exist, such as using Pearson’s correlation, partial correlation or mutual information of time patterns [1], which are typically calculated between the mean time series of brain regions.

In the last few years, DWI and fMRI data have been combined to study multimodal aspects of brain connectivity [6]. DWI is typically used to study white matter, whereas fMRI focuses on grey matter. The roles of grey and white matter in the brain have long been studied separately, but their interaction with each other is essential in the function of the brain. The study of functional and structural connectivity, and especially how they relate to each other, could help to better understand the roles and interaction of grey and white matter. Structural and functional connectivity have been combined both to model their relationship [7] and to derive new types of connectivity [8]. Wee et al. (2012) [9] built a machine learning model using structural and functional connectivity to determine which subjects would develop mild cognitive impairment. With an increased interest in multimodal brain connectivity analysis, it is likely that many more publications on the subject will appear in the coming years.

As the study of multimodal group comparisons progresses and new types of connectivity are developed, researchers require methods to evaluate which connectivity metric is best suited to their research question, e.g. to make an informed decision about which network types to include in their study, or to study the relation between different network types and make inferences about potential biological explanations for the observed differences and similarities. There is a need for methods of comparing functional and structural connectivity with respect to their suitability for group analyses, both uni-modally, and bi-modally.

An integral component of such analyses is visualization of results. Margulies et al. (2013) [10] conducted an in-depth review of uni- and multi-modal visualizations used in brain connectivity research, including an explanation of the benefits and drawbacks of each. Networks of connections between ROIs are often depicted in a matrix representation [11–13], where each row and column correspond to an ROI, and each element in the matrix represents a connection between the two (see S1A Fig). The same information can be displayed in a connectogram [10,14], which is a circular representation in which nodes are aligned along the edge of a circle and connections are represented by lines between them (see S1B Fig). Both representations have benefits and drawbacks. For example, the matrix representation is able to show all possible connections simultaneously, however they are an abstract way of representing connectivity and the focus on connections may result in understating or not detecting important individual ROIs. Connectograms, on the other hand, are well-suited to draw attention to important nodes and they represent connections in an intuitive way, namely by connecting two ROIs by a line; however they are limited in the number of connections that they can effectively show at the same time. Connectivity is also frequently shown within a three-dimensional (3D) representation of the brain [7,12,13,15,16], where each ROI is represented as a point in 3D space and a connection is depicted as a line between two points (see S1C Fig). While these visualizations can be impressive, they are also limited by the number of connections that they can display without obscuring valuable connectivity information [10] and the depiction of these 3D objects in two-dimensional images results in ambiguity about the position of each node in the third dimension. Relationships between structural and functional connectivity have previously been presented using scatter plots [7] (see S1D Fig). However, a network often contains hundreds or thousands of connections. When displayed together in the same scatter plot, overlapping points make it difficult or impossible to know the density of points at given position in the plot. Additionally, when two groups are displayed within the same scatter plot, the points of one group may obscure the view of points from the other group, making it impossible to know where the groups overlap and where they do not.

The aim of this study is to enable multi-level comparison, integrated analysis and visualization of multiple functional and structural connectivity types, where connectivity is defined between pairs of ROIs. Our contribution is three-fold. First, we propose a method to enable both uni- and bi-modal analysis of structural and functional data. Second, we present two proof-of-concept studies by comparing groups of middle-aged to elderly subjects and patients with schizophrenia to controls. Third, we propose a set of visualizations that address some of the above-mentioned issues in the context of an analysis of group differences in structural and functional connectivity.

## Material and Methods

**Fig 1:**
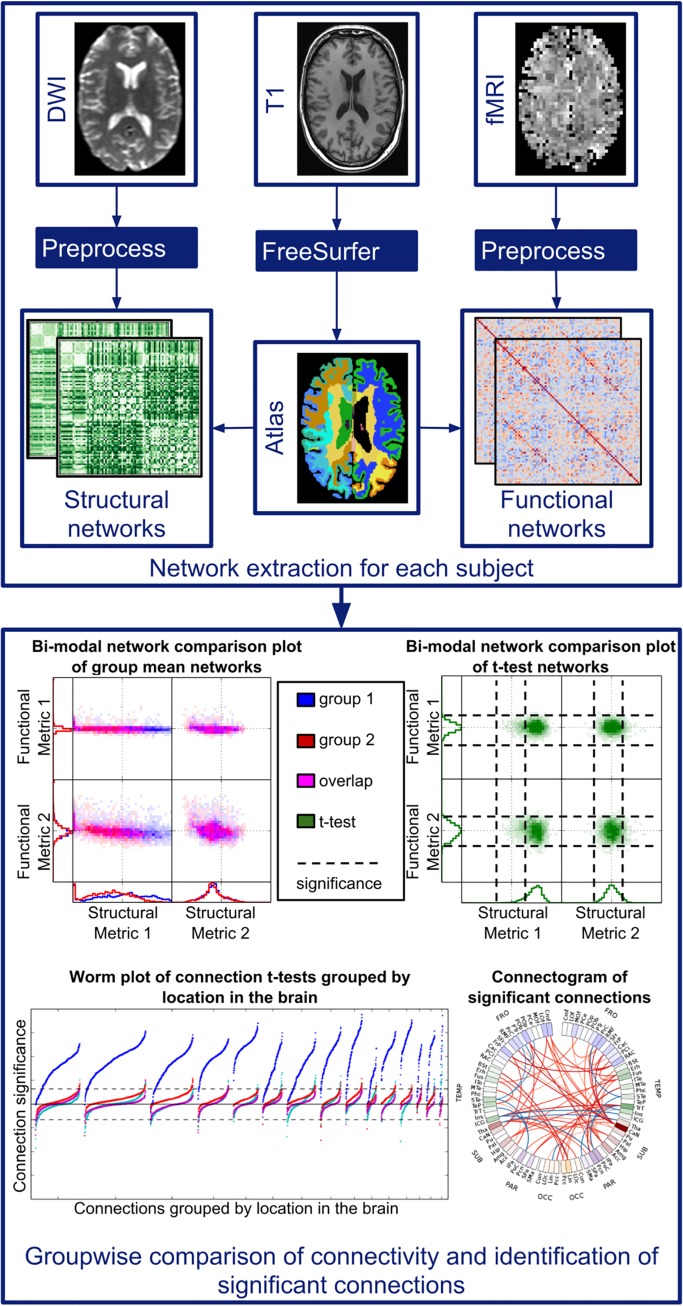
Schematic of method. Networks are extracted for each individual (upper panel) by extracting brain regions from the T1 image using FreeSurfer, and extracting structural and functional connectivity between these regions from preprocessed DWI and fMRI, respectively. Subsequently, integrated analysis of group differences in structural and functional connectivity is performed (lower panel). Group differences are visualized on a global level using bi-modal distribution plots, on an individual connection level using connectograms and on a mid-level using worm plots, where connections are grouped by location in the brain.

The proposed method to perform integrated analysis of functional and structural connectivity is illustrated in Fig 1. Each subject requires a DWI, fMRI and structural T1-weighted image. First the T1 image is segmented into a set of regions using FreeSurfer [17], after which structural and functional connectivity maps are extracted. The connectivity maps of each subject are compared uni- and bi-modally for their utility in group-wise analysis as well as identification of connections that contribute to group differences and similarities. The methods are applied to two proof-of-concept studies, one that compares groups of middle-age and elderly subjects, and the second that compares patients with schizophrenia to controls. More details of these steps are provided below.

### Segmentation of cortical ROIs

Cortical reconstruction and volumetric segmentation of each subject’s T1 image to produce an ROI atlas was performed using the FreeSurfer image analysis suite [17], available online at http://surfer.nmr.mgh.harvard.edu. The atlas was registered with 12 degrees of freedom to both the DWI’s b0 image and a mean fMRI, also using FSL’s FLIRT algorithm.

### Extraction of structural connectivity

The DWI's were preprocessed using AFNI and FSL tools [18]. First they were corrected for motion and Eddy currents and the brain extraction tool was used for skull stripping of the Eddy-corrected DWI. Duplicate b0 volumes were merged into a single volume by taking the median across duplicate images at each voxel. FA and mean diffusivity (MD) images were generated from the skull-stripped DWI using DTIFit. All three images (DWI, FA and MD) were masked using a mask generated from the registered FreeSurfer image, including cortex, white matter and subcortical regions.

The network extraction step of the “Statistical Analysis of Minimum cost path based Structural Connectivity” (SAMSCo) framework [4] was used to define structural connectivity because it minimizes the influence of directional uncertainty while finding globally optimal paths. A minimum cost path network (DMC) was extracted, with nodes defined by ROIs in the registered atlas and edges defined as minimum cumulative cost to travel between ROIs. A second metric of structural connectivity, the average fractional anisotropy (DFA) network, was produced by dividing the sum of fractional anisotropy along a minimum cost path by the Euclidean distance of the path. SAMSCo produces two values per connection, one from the seed to target region and the other from the target to seed region. Therefore, the SAMSCo networks were symmetrized by choosing the minimum of the two costs (and corresponding DFA values) to represent the edge between each pair of regions. It is important to note that SAMSCo is a less traditional choice of structural connectivity measure, an aspect which is discussed in more detail in the “Image preprocessing and network generation” subsection of the Discussion section.

### Extraction of functional connectivity

Preprocessing of the fMRI's was accomplished using ANFI and FSL tools [18,19]. The fMRI images were slice-timing and motion-corrected, both using AFNI tools. The first four TRs were removed to allow for equilibration and a temporal 0.01Hz high pass filter was applied to remove low frequency signals. Global mean, mean cerebral spinal fluid, and mean white matter time series as well as the six motion parameters resulting from motion correction were regressed out from the image. Finally, an 8mm full-width-half-maximum Gaussian spatial filter [20] was applied to increase the signal to noise ratio.

We defined two atlas-based metrics for defining functional connectivity. Typically, functional connectivity studies include a preprocessing step where fMRI images are spatially normalized to a common reference space and blurred to account for differences in structural and functional variability. However, anatomical and functional variation between subjects can be large [20], and thus the deformations involved in spatial normalization may stretch or contract parts of the brain for some subjects more than others. In this study, spatial normalization was not performed. Rather, the FreeSurfer ROIs were registered to the fMRI image for each subject separately, and then used to define connectivity. This reduced the effects of structural variability.

The average time series for each ROI was calculated. For the first network type, connectivity between two ROIs was defined as the Pearson correlation coefficient between each ROI's mean time series (fMT). For the other type of connectivity we used partial correlation (fPC), which was calculated for every ROI pair with the influence of all other ROI’s removed according to the formula $r_{ij}=-p_{ij}\;/\sqrt{p_{ii}p_{jj}}$ [21], where *p*_*ij*_ is the *(i*,*j)*th element of the inverted covariance matrix from the mean time-series data of all ROIs.

### Integrated analysis and visualization of structural and functional networks

An ordinary least squares linear regression model was estimated for each connection including group membership, modeled as a single variable containing zeros and ones, and covariates as variables, along with a constant term to model the mean of the data. The t-statistic and p-values associated with group membership were used to determine whether a connection’s weight differs significantly between groups. The group mean of each connection was also computed for each group of subjects. The t-statistic and group means were visualized using three types of visualizations, illustrated in the lower panel of Fig 1. These include bi-modal network comparison plots of group mean networks and t-statistic networks, worm plots and connectograms. This section provides an explanation of each. All of the visualizations presented in this manuscript can also be used to study continuous variables rather than group differences, which is discussed in more detail in the Discussion section.

Bi-modal network comparison plots enable simultaneous evaluation of multiple network types on a global level, both uni- and bi-modally. As seen in Fig 1, they can show distributions of group mean connection weights from two groups simultaneously, or distributions of t-statistics, which illustrates degree of group differences on a global level. Each type of connectivity is represented by a column or row, where rows represent the functional measures and columns represent structural measures. Each connectivity type is evaluated uni-modally in one-dimensional (1D) histograms. The relationship between structure and function is shown in two-dimensional (2D) histograms for each pair of structural/functional network types, which is displayed in the center of the plots. In the case of the group mean networks, colors are used to differentiate between groups, where blue represents one group and red represents the other. In the 2D histograms of group mean networks, magenta indicates group overlap. In the plot of the t-statistics, green is used to represent group differences.

Worm plots show the connection t-statistics grouped by the location of the associated ROIs. Even when very few connections are individually identified as significant, the worm plot may show clusters of connections that have significant group differences when considered together (regional shifts in connectivity between multiple ROIs). Significance is thus evaluated on two scales, namely on the level of individual connections and on the level of groups of connections. Worm plots are a derivative of Manhattan plots, which are scatter plots used to show significance of a large number of tests, most of which have very large p-values. They are frequently used in genetic studies [22], where genomic coordinates are on the x-axis and colors of points represent groupings of points. A few key changes to the Manhattan plot were made to make it more suitable to brain connectivity analysis. First of all, connections were clustered by the parts of the brain to which its ROIs belong. Unlike in genetic studies where base-pairs are ordered based on their position within DNA, in the brain there is no intrinsic ordering of connections within each cluster, therefore each cluster was ordered according to t-statistic values. This reordering gives an indication of the distribution of points within each cluster, and enables simultaneous plotting and comparison of multiple network measures. Normally the y-axis of a Manhattan Plot is −log_10_ (*p*) where *p* is the p-value corresponding to a statistical test. We instead used $-{\text{log}}_{10}\left(p\right)*sign\left(t\right)*s_{0.05,M_{eff,G}}$, where *t* is the t-statistic of a given connection, *p* is the corresponding p-value and $s_{\alpha ,M_{eff,G}}={\text{log}}_{10}\left(\alpha \right)/{\text{log}}_{10}\left(\alpha_{M_{eff,G}}\right)$ is a scaling factor used to ensure that the reference line corresponding to the adjusted significance threshold, $\alpha_{M_{eff,G}}$ (see below for details of its derivation), is the same for all networks *G*. *sign*(*t*) adds information about whether an observed association is positive or negative. This, combined with ordering connections, resulted in clusters of points with a worm-shape, hence the name *Worm Plot*. The distance of the mean of each cluster from zero indicates the degree to which the associated parts of the brain play a role in the relationship being tested.

Significant connections for each network were also plotted in a connectogram, which was originally used to study brain connectivity by van Horn et al. (2012) [14]. The 2D circular representation in a connectogram provides a cleaner view of brain connectivity than 3D representations. While 3D representations can provide spatial information about ROIs [7,12,13,15,16], viewing them requires projections onto a 2D space. The loss of one dimension results in ROIs appearing spatially closer than they actually are. For example, when viewing a 3D representation axially, the frontal pole and lingual gyrus appear to be located beside each other, when in reality they are on opposite sides of the brain. This also presents difficulties in interpreting region groupings, since groups which are spatially separate will likely appear to overlap in the projected view. The connectogram removes these complications by disregarding 3D spatial information. In the circular representation, ROIs are arranged on the edge of the circle, grouped into the same clusters as in the worm plots. Each cluster is assigned a color, and the concentration of color is adjusted according to node degree. Significant connections are represented as lines between ROIs. The color of each significant connection is determined by the sign of the t-statistic and the opacity indicates the degree of significance.

Both worm plots and connectograms require a threshold of test significance. Given that a t-statistic is computed for each connection, some form of multiple testing correction must be used. In the case of a symmetric network *G* with *r* regions, the total number of tests is *M*_*G*_ = *r*(*r*−1)/2. Traditional methods, such as the Bonferroni and Šidák methods, assume independence of tests and calculate a new significance threshold from the total number of tests. However, the connected nature of brain networks implies dependence between connections. As such, traditional methods are overly conservative for connectivity analysis. To address this, we used a method proposed by Li et al (2012) [23], where an “effective number of independent tests” for network *G*, *M*_*eff*,*G*_, is calculated. *M*_*eff*,*G*_ replaces *M*_*G*_ in the traditional methods. *M*_*eff*,*G*_ was applied to the Bonferroni correction to calculate an adjusted p-value threshold, $\alpha_{M_{eff,G}}=\alpha \;/\;M_{eff,G}$. We used *α* = 0.05 as the uncorrected threshold.

### Proof-of-concept

Two proof-of-concept studies were performed using the analysis described above. The first compared middle-age and elderly subjects from the Rotterdam Scan Study (RSS) [24,25]. The second compared patients with schizophrenia to healthy controls from the Massachusetts General Hospital (MGH) in the MIND Clinical Imaging Consortium data collection [26], which is an open-access multi-site collaborative study of patients with schizophrenia. The original MGH imaging data and subject meta-data is available for download via http://coins.mrn.org/dataexchange. The networks calculated for each subject and the regression results calculated as part of this study are available at http://dx.doi.org/10.5061/dryad.88q04.

There were 84 and 82 cortical and subcortical regions chosen for network construction for the MGH and RSS studies, respectively. The cerebellum was excluded in the RSS sample because it was only partially scanned. After registration of the FreeSurfer segmentation to the diffusion image space, which required up-sampling of the data, two regions of one subject in the RSS study did not have any voxels remaining. Regional statistical tests involving these regions were computed by substituting group means for the missing values. See S1 Table for a list of regions used in these studies.

In the RSS data, after registration and down-sampling of the FreeSurfer atlas to the fMRI-space, in two regions of one subject no voxels survived the down-sampling (the left entorhinal cortex and the right temporal pole). All analyses did not include the missing values.

#### Participants

In the RSS study, non-demented subjects with T1, rs-fMRI and DWI scans were divided into two groups (middle-aged and elderly) based on age. Age ranges were 74 to 95 in the elderly group and 51 to 53 in the middle-aged group. They were selected such that the gender ratio in each group was the same. Subjects with mild cognitive impairment (as determined by the Mini-Mental State Examination [27], MMSE), large infarcts, and gliomas were excluded from the study. The MMSE score is a derived measure of a subject’s global cognitive functioning with a total possible score of 30 [27].

In the MGH study, groups of patients with schizophrenia were compared to controls. Exclusion criteria included an intelligence quotient less than 70, a history of significant head injury and a contraindication for MRI scanning. Only subjects who had DWI, fMRI and pre-computed FreeSurfer segmentations were included. Parental socio-economic status (P-SES) was defined by the Hollingshead-Redlich scale [28], in which a score of one defines the highest status and five defines the lowest. P-SES is often used as an estimate of what the socio-economic status of patients would have been if they did not have schizophrenia. Years of education is defined as the total number of years that a subject pursued elementary, secondary and post-secondary education.

In both studies, subjects with more than 4mm of head movement were excluded. All participants gave written informed consent and the original studies were approved by the medical ethics committee of the Erasmus MC, University Medical Center Rotterdam (for the RSS study) and the institutional review board of the Massachusetts General Hospital (for the MGH study). Table 1 describes the demographics of each group for the subjects that were included.

**Table 1:** Subject demographics.

Study	RSS			MGH		
Group	Middle-aged	Elderly	Group difference p-values	Patient	Control	Group difference p-values
No. of subjects	37	37		23	21	
Gender (M/F)	13 M / 24 F	13 M / 24 F	0 ^χ^	17 M / 6 F	12 M / 9 F	0.9 ^χ^
Age (mean ± SD)	52.3 ± 0.6	81.1 ± 4.4	<0.0001 ^t^	34.8 ± 9.8	38.7 ± 8.7	0.2 ^t^
MMSE (mean ± SD)	28.9 ± 1.1*	27.1 ± 1.7*	0.002 ^t^	n/a	n/a	n/a
Years of education (mean ± SD)	n/a	n/a	n/a	11.5 ± 2.2	15.9 ± 2.0*	<0.0001* ^t^
P-SES (mean ± SD)	n/a	n/a	n/a	3.3 ± 1.0	3.0 ± 1.0	0.9 ^χ^
fMRI absolute head motion in mm (mean ± SD)	1.0 ± 1.0	1.0 ± 0.9	0.9 ^t^	0.4 ± 0.4	0.6 ± 0.6	0.4 ^t^

* One subject was missing this information, statistics computed over the remaining subjects

^t^ Calculated using Welch’s t-test

^χ^ Calculated using χ^2^ test

#### Image acquisition

All subjects from the RSS were scanned on the same 1.5 Tesla GE scanner with an 8-channel head coil. A research physician visually inspected each scan to ensure good quality. The T1-weighted protocol included a 3D fast RF spoiled gradient recalled acquisition in steady state with an inversion recovery prepulse sequence (TR = 13.8 ms, TE = 2.8 ms, TI = 400 ms, FOV = 21 × 21 cm^**2**^, matrix = 416 × 256, zero-padded to = 512 × 512, flip angle = 20°, NEX = 1, bandwidth = 12.50 kHz) with 96 contiguous slices with slice thickness of 1.6 mm zero-padded to 0.8mm. The final voxel size was 0.49 × 0.49 × 0.8 mm. The DWI protocol utilized a single shot, diffusion-weighted spin echo-planar sequence (TR = 8000 ms, TE = 68.7 ms, FOV = 21 × 21 cm^**2**^, matrix = 96 × 64, zero-padded to 256 × 256, NEX = 1) with 36 contiguous slices with 3.5 mm slice thickness. The maximum b-value was 1000 s/mm^**2**^ in 25 non-collinear directions. Volumes were also acquired without diffusion weighting (b0). The final voxel size was 0.8 × 0.8 × 3.5 mm. The rs-fMRI protocol included a gradient-echo BOLD sequence, (TR = 2900 ms, TE = 60 ms, matrix = 64 × 64, flip angle = 90°) with 35 contiguous 3.3 mm slices and 160 volumes (total scan length = 464 s). The voxel size was 3.3 × 3.3 × 3.3 mm.

The MGH subjects were scanned on a 3 Tesla Siemens Trio scanner with an 8-channel head coil. The imaging parameters of the T1-weighted sequence were TR = 2530 ms, TE = 3.79 ms, TI = 1100 ms, FOV = 16 mm, matrix = 256×256×128 cm, flip angle = 7°, bandwidth = 181, 0.625×0.625 mm voxel size and slice thickness 1.5 mm. The DWI scan parameters were TR = 10,500 ms, TE = 98 ms, NEX = 2, bandwidth = 1342, 64 slices, thickness = 2 mm, 2×2 mm voxel size. The maximum b-value was 1000 s/mm^2^ in 12 directions. The fMRI parameters were TR = 2000 ms, TE = 30 ms, flip angle = 90°, FOV = 22 cm, bandwidth = 3126Hz/pixel, 27 slices, slice thickness = 4 mm with 1 mm skip, 3.4 mm in plane resolution and 177 volumes (total scan length = 354 s). Three runs of the Sternberg item recognition paradigm (SIRP) [29] were collected. SIRP is a task-based fMRI sequence with a block design that measures working memory. The run with the least amount of motion was selected for connectivity analysis.

#### Covariates

Covariates were included in the regression model to control for their influence. In the RSS study covariates included MMSE and gender. In the MGH study covariates included age and gender.

## Results

The chosen connectivity metrics were evaluated both uni- and bi-modally described in the methods section. The resulting figures and tables are described below for both the MGH and RSS studies.

**Fig 2:**
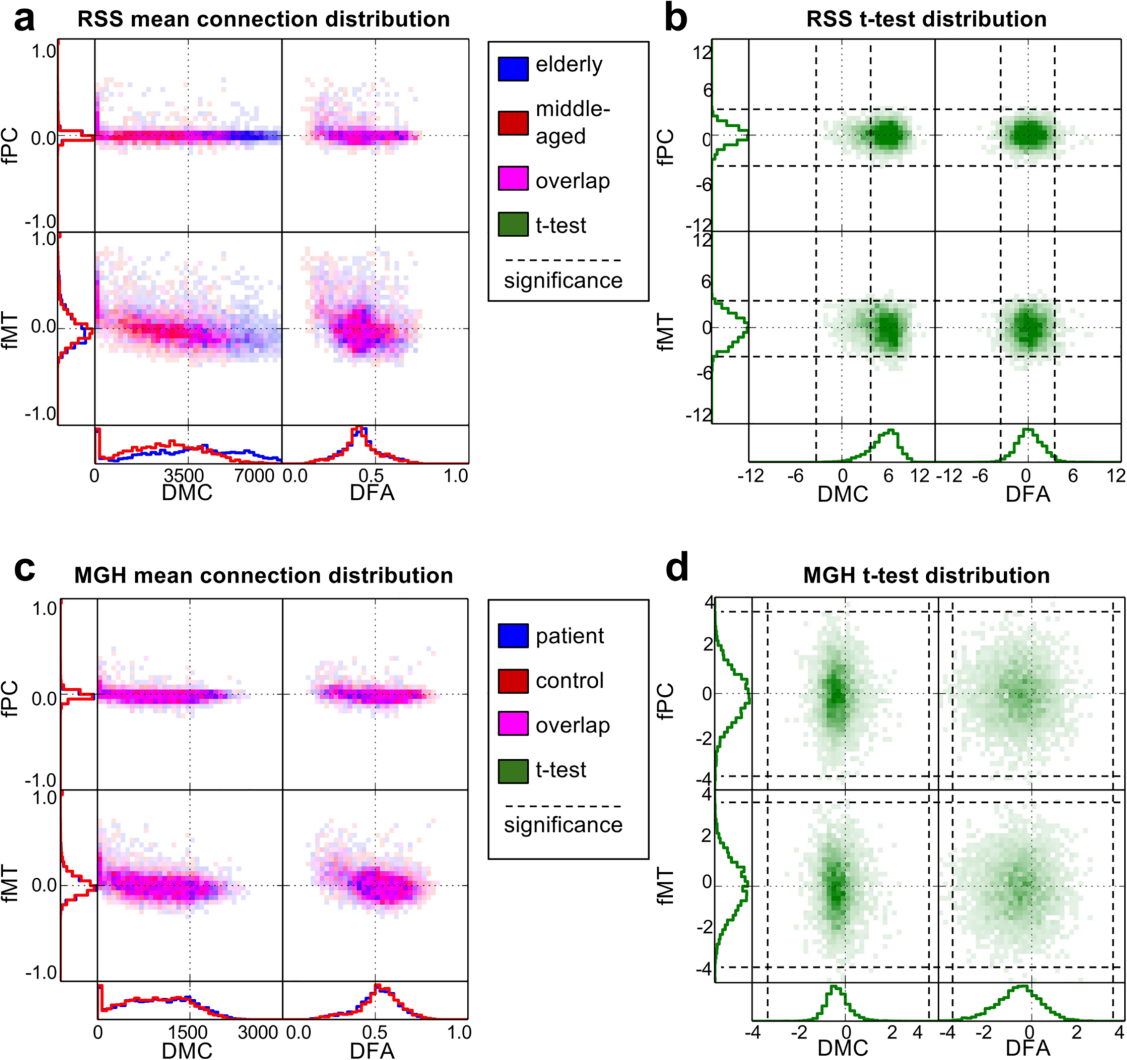
Bi-modal network comparison plots. Structure-function relationships are shown for the mean connectivity network of each group in (a) and (c), and the distribution of t-statistics in (b) and (d). (a) and (b) correspond to RSS, where in (a) blue represents the elderly group and red indicates the middle-aged group. (c) and (d) correspond to MGH, where in (c) blue represents the patients with schizophrenia and red represents controls. In the 2D structure-function histograms, magenta indicates overlap of the two groups. In the t-statistic histograms, green shows group differences, where positive values indicate elderly > middle-aged in RSS and schizophrenia > control in MGH.

The bi-modal network comparison plots in Fig 2 show the structure-function relationship for the mean networks and t-statistic distributions for each study. 2D histogram opacity indicates number of connections, and in the mean network plots, color indicates the proportion of each group involved. Along the axes, distributions of connection weights for each individual connectivity type are plotted.

In the RSS study, group differences are easily visible in the mean network histograms of DMC, which is also reflected in the t-statistic distributions. Global group differences are not obvious in the mean network plots or the t-statistic plots for fPC and fMT in both groups and DFA in the RSS. In the MGH study, global group differences are not obvious in the mean network plots, but do show in the t-statistic plots for DMC and DFA.

Both studies show a large peak in mean DMC near zero. In order to determine whether this peak is fully explained by connections between adjacent regions we regenerated the histograms after omitting all connections between adjacent regions. In the MGH study, the peak disappeared. In the RSS study the peak did not fully disappear, but did become much smaller.

In the unimodal t-statistic histograms, all types of connectivity appear to be approximately normally distributed. The positive shift in t-statistic of DMC in the RSS study indicate a global increase in minimum cost in the elderly population. In the MGH study, there is a global decrease in DMC and DFA. It is interesting to note that both fMT and fPC mean network values were centered around zero and the standard deviation of fPC was much smaller than fMT in both studies, but the t-statistic indicated similar levels of group differences. The 2D t-statistic histograms show no significant correlation between structural and functional t-statistics.

**Fig 3:**
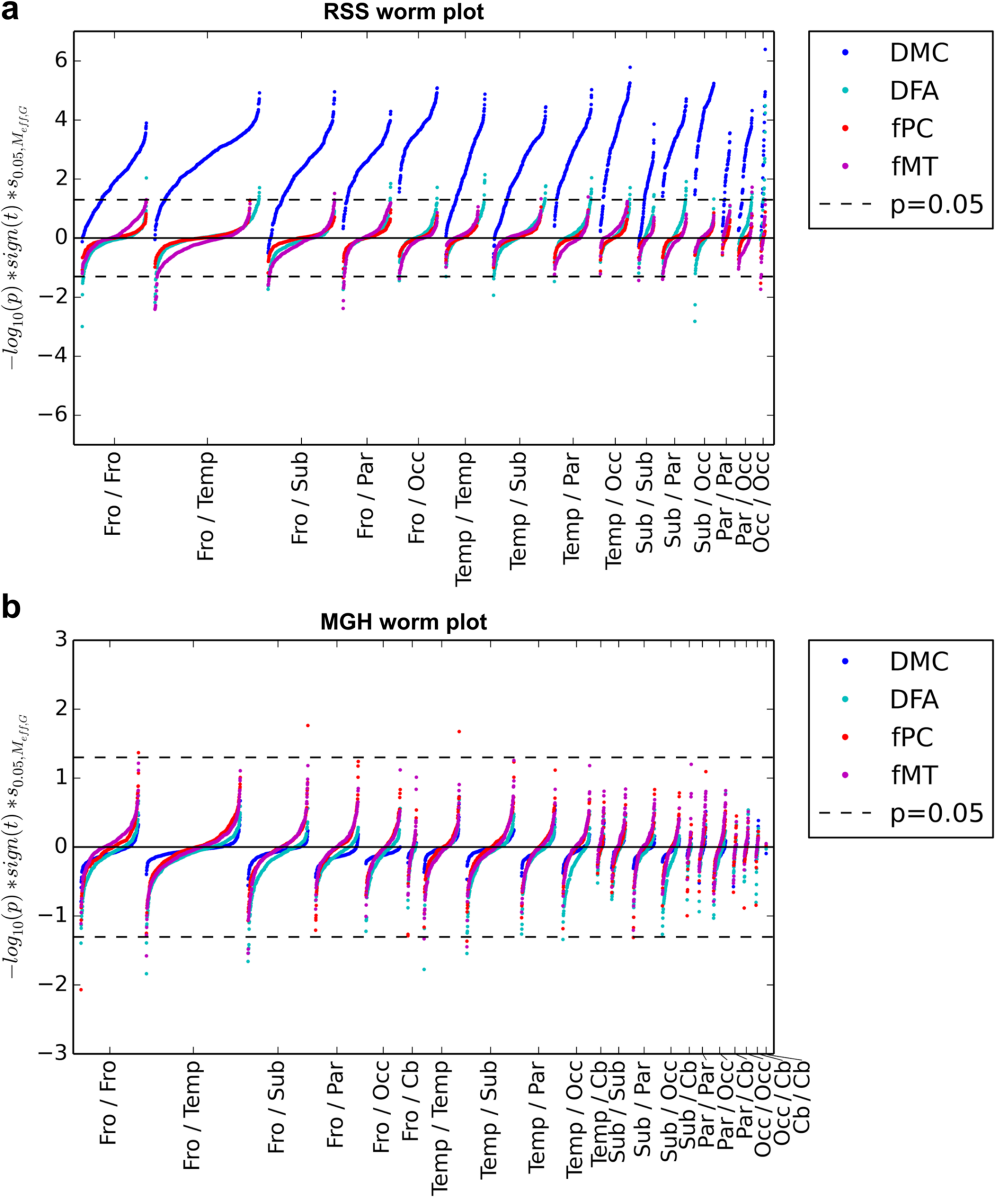
Worm plots. Connections in the (a) RSS and (b) MGH studies are grouped by location of region pairs. Groupings include subcortical (Sub), occipital (Occ), parietal (Par), temporal (Temp), frontal (Fro) and the cerebellum (Cb). Cb was not included in the RSS study. On the y-axis is the negative log of the p-value, multiplied by the sign of the corresponding t-statistic and scaled such that the line indicating p = 0.05 is at the same position for all plots.

A worm plot of the p-values corresponding to the t-statistics of each connection is shown in Fig 3. The regions of the brain are divided anatomically into groups corresponding to different parts of the brain, including subcortical, occipital, parietal, temporal, frontal and the cerebellum. The cerebellum was not included in the RSS study because it was only partially scanned.

In the RSS study, DMC has many highly significant connections, with values well above the significance level for the majority of connections in all regions (Fig 3A). In the MGH study, very few connections show significant group differences. In both studies, some networks are shifted away from −log_10_ (*p*) = 0 for some clusters. T-tests were done on the t-statistic values of the connections in each worm in Fig 2 to determine the degree to which they were shifted away from *t* = 0, which is visualized in S2 Fig.

In the RSS worm plot, it is obvious that all clusters except Fro/Temp have highly significant positive DMC associations with age. The shifts in DFA and fMT are weaker than those of DMC and are mixed between positive and negative connections. Several significant cluster shifts appear in both. There were no significant fPC cluster shifts.

In the MGH study, DMC and DFA had the most significant associations with schizophrenia, all of which were exclusively negative. fMT and fPC had a mixture of positive and negative associations which were much weaker than the structural connectivity associations. fPC had particularly weak associations.

**Fig 4:**
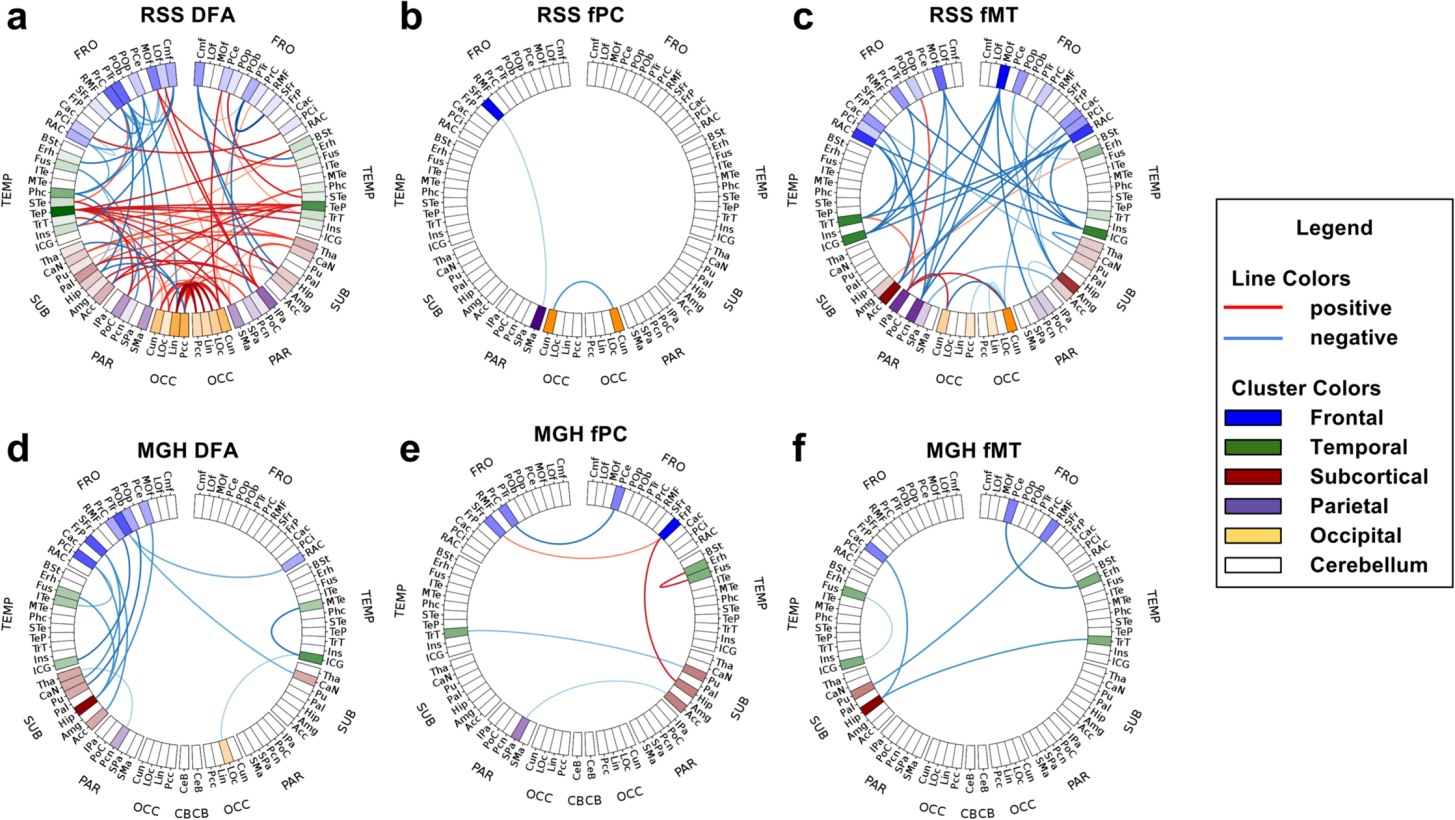
Connectograms of significant connections. Red connections have significantly positive t-statistic values corrected for multiple testing, blue are negative (in the MGH schizophrenia > control, in the RSS old > young). Degree of significance is indicated by the opacity of the lines. Color saturation of regions shows the number of connections involving that region. For the RSS (a) DFA, (b) fPC and (c) fMT are shown. RSS DMC is not shown because there were too many significant connections to visualize. For the MGH (d) DFA (e) fPC and (f) fMT are shown. MGH DMC is not shown because there were no significant connections.

The connectograms in Fig 4 show the significant connections of some of the networks for each study. Red indicates significant connections with positive association (in the MGH this implies schizophrenia > control, in the RSS old > young), and blue indicates negative association. The opacity of the lines indicates the level of significance and the saturation of each region indicates the number of significant connections involving that region. In the RSS study, DMC is not shown because it had too many connections to extract meaningful conclusions. In the MGH study DMC is not shown because it had no connections with $p<\alpha_{M_{eff.G}}$.

In the RSS study, the DFA network had several significant connections with both positive and negative associations with age. The positive associations were concentrated in the temporal and occipital lobes. The left temporal pole had many more significant connections than the other regions. The right temporal pole also had several significant connections. Inter-hemispheric significant DFA connections were exclusively positive, indicating an increase in the older group. Decreases in DFA were exclusively intra-hemispheric, many of which involved left frontal regions. There were three significant RSS fPC connections, all of which were intra-hemispheric. The RSS fMT connectogram shows exclusively negative associations, many of which involved the right medial orbitofrontal cortex.

In the MGH study, all significant DFA connections indicated negative associations with schizophrenia, most of which involved only the left hemisphere and especially frontal and temporal regions. MGH fPC connections were both positive and negative. All three negative fPC significant connections were inter-hemispheric. All five significant fMT connections were negative. Three of them involved frontal regions.

## Discussion

This study presents a framework for comparison of ROI-based functional and structural connectivity to identify group differences. Given two groups of subjects, each of which has T1, DWI and fMRI images, networks were constructed, and then compared uni- and bi-modally for their ability to explain group differences. The t-statistic based analysis of each connection identified which network types contribute most to group differences, and between which areas of the brain these differences occur.

There are a growing number of types of structural and functional brain connectivity available. The methods described in this paper can be used to compare multiple types of connectivity, both in uni-modal studies and for integrated analyses. Visualizations of significance of connections help to identify important connections and groups of connections and compare them to existing knowledge of group differences. With the proposed framework two proof of concept studies were conducted comparing structural and functional connectivity.

### Bi-modal network comparison plots

The bi-modal network comparison plots in Fig 2 give important insights into the investigated network types and the relationships between them. They simultaneously show both uni- and bi-modal distributions, which makes it easy to consider both at the same time. Viewing all connectivity types in the same plot consolidates the information, making comparisons easier.

Global group differences can sometimes be seen in the 1D histograms of each network type. For example, in the mean-network plot of the RSS, DMC obviously has group differences, indicating that elderly subjects have higher cost paths than middle-aged subjects. Even when global group differences were not obvious in the mean-network plots, differences were sometimes found using the t-statistic plots. For example, in the MGH, DMC and DFA showed no obvious group differences in the mean-network plots, but did show global negative shifts in the t-statistic plots, suggesting that patients with schizophrenia have lower cost paths with reduced FA. The decrease in DFA is consistent with previously observed white matter abnormalities [30] and decreased DFA [31] in patients with schizophrenia. The decrease in minimum cost in the schizophrenia group is unexpected, but might be explained by the inclusion of age as a covariate. As can be seen in the RSS DMC histograms, DMC is highly influenced by age. Patients with schizophrenia were on average older than controls. Including age as a covariate would have removed the effect of age differences, but it is possible that the regression overcompensated for this variable and that the global decrease is not representative of the true role of minimum cost in schizophrenia. To determine the true effects of schizophrenia on DMC, the analyses should be repeated on an age-matched sample. Both mean-network and t-statistic plots provide useful information when examining connectivity metrics in relation to group differences.

The 2D part of the mean-network plots show bi-modal relationships between connectivity types. In both studies, the patterns seen in the mean-network plot show a similar pattern, indicating that the mechanism governing the global functional and structural differences may be inherent to the connectivity metrics themselves, and not related to the subject groups being studied. For example, both fPC and fMT are increased where DMC is near zero. DMC is usually near zero between adjacent regions because there is no distance between them, and hence minimal travel costs. This close proximity may also explain the increase in functional connection weight, since adjacent regions likely have similar blood-oxygen dynamics near their border and hence greater correlation.

The bi-modal view can also be used to make inferences about differences in connection types from the same modality. For example, global group differences are much lower in magnitude in fPC than in fMT. The difference in the derivation of fPC and fMT is that fPC uses partial correlation to estimate the direct connection between specific regions [32], whereas fMT uses full Pearson correlation. Thus, global group differences are greater in indirect connections than in direct connections, which may affect sensitivity of the metric to specific effects. fPC and fMT are both well-established widely used connectivity metrics, and the choice of which to use depends on the research question one wishes to answer [21].

We decided to represent the bi-modal part of the network comparison plots with 2D histograms. The relationship between structure and function can also be visualized using scatter plots [7]. While the same general trends can be seen in histograms and scatter plots, in places with high density, points in scatter plots will overlap and thus be not differentiable, in which case scatter plots are unable to show differences in density. By using a histogram, differences in density can be shown using color density. Another disadvantage of scatter plots is that overlap of points makes seeing group differences difficult. When points from one group cover points from another group, it may appear as though only one group is important. 2D histograms can better represent the degree of each group’s influence by representing each group by a given color, where the degree of overlap is indicated by combining the two colors. This is seen in the RSS mean-network plot, where the elderly subjects are represented by blue, the middle-aged subjects by red and overlap is represented as magenta. It is clearly visible that high DMC is dominated by elderly subjects, and low but non-zero DMC is dominated by middle-aged subjects. Moderate and near-zero DMC is equally represented by both groups. Group visualization is in most cases, however, limited to two groups since more colors would likely make it harder to differentiate where each group has influence. The bi-modal network comparison plot could also be used with continuous variables. For example, age can be modelled continuously rather than using age groups. In this case, rather than computing group mean networks, one could simply make 1D and 2D histograms of structural and functional weights over all subjects, but color each part of the histogram according to the mean age associated with all points in a given connection weight range. If a gradient of color is seen, then this indicates that connectivity changes globally with age. Rather than plot the t-statistic related to group differences, one could plot the regression coefficient associated with age.

The data presented here indicated no global functional group differences, neither in 1D nor in 2D. However, with other data the 2D histograms might be useful in identifying important group differences involving both functional and structural connectivity. It is possible that little or no group differences are obvious uni-modally, but that they become obvious when viewed bi-modally. The connectivity types used here also did not show an obvious relationship between functional and structural t-statistics, however if such a relationship did exist, the 2D representations would help to see it. Such relationship would indicate that connections with significant structural differences tend also to have strong functional differences.

### Worm plots

The worm plots in Fig 3 give important insights into regional changes in connectivity. Worm plots reflect global changes in connectivity, but often also show cluster level changes. The global changes in DMC in both studies and in DFA in the MGH study were also seen in the worm plots by the uni-directional shifts of each group of connections. All clusters in the worm plots were shifted to different degrees, indicating a relative importance of each cluster. Even when global network changes were not observed, shifts in regional groupings of connections became apparent in the worm plots. fPC and fMT had positive and negative shifts for both studies, as did DFA in the RSS. fMT and DFA both had large shifts, whereas fPC cluster shifts were minimal. This suggests that fPC is less sensitive to group differences than the other metrics. As in the bi-modal network comparison plots, this reflects the removal of the influence of other regions in a given correlation. A thorough psychological analysis of group differences identified in the worm plots is beyond the scope of this study and is limited by small sample sizes. However, we believe that epidemiological studies with high statistical power would benefit from analysis using worm plots.

As with the bi-modal network comparison plots, the worm plot can be used to represent continuous variables rather than groups. For example, if age was modeled continuously, one could simply replace the sign of the t-statistic in the y-axis with the sign of the coefficient associated with age. As with age groups, the continuous version of the plot would show the association of age with grouped connections.

If one was to judge a network’s ability to separate groups purely based on global network differences and individual significant connections, it would be easy to dismiss a network as insensitive to group differences. However when looking at regional groupings of connections it may become apparent that group differences do exist. The worm plot provides a mid-level analysis, more specific than the global differences seen in the bi-modal network comparison plots, but more general than looking only at specific significant connections. The worm plot extracts important relationships that are otherwise invisible to global or individual connection based analysis.

### Connectograms

The connectograms in Fig 4 visualize significant differences in specific connections in a 2D representation, where nodes are grouped by location in the brain. This enables quick identification of individual significant connections, significant groups of spatially-related connections and nodes with many significant connections. In both the RSS and MGH studies, the connectograms provided a clear and easily interpretable representation of significant connections in the DFA, fPC and fMT networks. Again, similar to the bi-modal network comparison plots and worm plots, one can use connectograms to visualize the association of connections with a continuous variable by determining the color of a connection by the sign of the coefficient associated with it.

Nodes with many significant connections are immediately obvious in connectograms. For example the left and right temporal poles (TeP) in the RSS DFA connectogram and the right medial orbitofrontal cortex (MOf) in the RSS fMT connectogram are easy to spot because they have several highly significant connections associated with them. The left hippocampus (Hip) in the RSS DFA also has several significant connections associated with it, which can be spotted by the region’s increased color saturation, however it is less obvious because crossing connections make it difficult to see how many connections are coming from that region. Regions with many significant connections might be less obvious in a 3D representation of brain connectivity, depending on from which angle it is viewed. This would arise because a 3D representation must be viewed as a 2D projection, which might result in an important region being surrounded by unrelated connections and regions that are spatially far away, but appear in that orientation to be nearby. Additionally, 3D representations can potentially misrepresent the data. For example, if two nodes overlap in a specific orientation, they may be interpreted as a single node, with connections from both nodes extending from the same spot. This confusion can be avoided by using connectograms.

The use of connectograms also made it easy to spot networks that have many significant connections that are primarily uni- or bi-lateral. For example, most significant connections in the MGH DFA network are uni-lateral negative associations with schizophrenia in the left hemisphere, which is consistent with the reduction of left hemisphere dominance seen in schizophrenia [33]. Also, the RSS DFA network has an abundance of positive inter-hemispheric connections and negative intra-hemispheric connections, suggesting that aging is associated with increased FA along inter-hemispheric minimum cost paths and decreased FA along intra-hemispheric minimum cost paths. An interesting avenue of future research might be to reconstruct the minimum cost paths found by SAMSCo to see if this unexpected increase can be explained by differing minimum cost paths in the old and young subjects.

A limitation of the connectogram is that there is an upper limit to the number of significant connections that it can show. The RSS DMC network had so many significant connections that useful inferences could no longer be made using a connectogram. This issue is also a limitation of 3D representations of brain connectivity. In such cases, one might consider using a matrix representation [11–13]. In this representation the connections never overlap, and thus all connections can be shown simultaneously. The disadvantage of a matrix representation, however, is that regions with several significant connections might be less obvious, since to identify them one must scan an entire row or column of a matrix and count the number of significant connections.

### Methodological considerations

#### Image preprocessing and network generation

Subject motion is an important issue to address in any neuro-imaging study. This is especially so in functional connectivity studies. Head motion has a significant effect on connectivity. It increases connectivity estimates over short distances, decreases them over long distances, reduces modularity and can inflate the effect of age on connectivity [34]. When preprocessing our fMRI data, we addressed subject motion by regressing out six motion parameters. Alternative approaches include 24 motion parameter nuisance regression, targeting volumes affected by motion and using independent component analysis, all of which are thoroughly evaluated by Pruim et al. (2015) [35] for their ability to both remove motion-related artifacts and to preserve the signal of interest. Compared to other approaches, regressing out either six or 24 motion parameters only minimally reduced the impact of motion artifacts and was not very reproducible. However, using six parameters had minimal loss of temporal degrees of freedom. They also examined data scrubbing, which is a widely-used approach that removes volumes affected by motion [36]. Although this approach is effective in removing variations induced by motion, it also results in a significant loss of temporal degrees of freedom and is less reproducible than many of the methods based on independent component analysis [35]. Given that subject motion can confound studies by inducing group differences, it is important to carefully choose an appropriate method of dealing with motion. Global signal regression, which was employed in this study, can reduce motion-related group difference [36]. It can remove false variance present in resting-state functional connectivity data, but it has also been linked to a negative bias in correlations computed from fMRI data [36]. Because of this, its use in functional connectivity studies is controversial and future studies must carefully examine the risks and benefits before deciding whether to use it or not.

Regarding choice of structural connectivity measures, it is important to note that both measures (DMC and DFA) are products of the SAMSCo framework, which always produces a fully connected graph in which each pair of regions has an associated connection weight, even when no physical connection is present. This reflects the possibility that regions may communicate with each other by sending signals *via* intermediate regions. On the other hand, more traditional definitions of structural connectivity, such as those based on streamline tractography, represent direct connections only and as such result in sparse matrices where only direct connections have non-zero values [37]. It is important to keep in mind whether a structural or functional network measure represents direct or indirect connectivity, since this can have a profound impact on the interpretation of the results.

#### Defining regions of interest

An important consideration when building brain connectivity networks is the method used to segment the cortex and sub-cortex [38]. Segmentations are a macroscopic representation of microscopic neuronal architecture, the relationship between which is not fully understood. This makes the choice of scale and algorithm complex. This study used an anatomical segmentation of the cortex and sub-cortex into anatomical regions, which has also been done in previous studies [4,39,40]. It is important to note that anatomical segmentations are one of many potential segmentation strategies. Zalesky et al. (2010) [41] used random segmentations to show that the scale of segmentation (i.e. the size and number of segmented regions) and the angular resolution of DWI data affect the topological attributes of structural networks. Randomly segmented regions do not generally correspond to specific anatomical or functional regions, and thus may not be suitable to answer questions where regions should represent known areas of functional specialization. Cammoun et al. (2012) [42] found a compromise between anatomic and random segmentation by subdividing FreeSurfer regions into smaller regions on multiple scales. Both Zalesky et al. (2010) [41] and Cammoun et al. (2012) [42] acknowledge that while segmentation scale affects the outcome of network studies, both coarse and fine segmentations have benefits and drawbacks, and thus choice of scale should be made depending on the research question.

The segmentations discussed thus far do not use fMRI to delineate borders between regions. This may result in regions which are not functionally distinct. Several groups are working on methods of using functional data to segment grey matter. For example, Blumensath et al. (2013) [43] uses region-growing in fMRI data to find subject-specific functionally homogeneous parcels which are spatially contiguous and disjoint. The method was reproducible within subjects, however there was no correspondence between subjects’ segmentations, which precludes between-subject direct comparison of connectomes using such a segmentation. Shen et al. (2013) [44] used a group-wise clustering algorithm to produce functionally homogeneous and non-overlapping parcels that correspond across subjects, thus allowing between-subject comparisons. These methods, unlike the anatomical methods, produce segmentations that estimate functionally segregated regions.

The anatomical methods and the two functional methods discussed above produce ‘hard parcellations’ [45], where regions are represented by non-overlapping groups of contiguous voxels. In some cases, hard parcellations may not be an appropriate representation of true functional parcellations and connectivity patterns. Previous studies have suggested that multiple overlapping functional networks exist [32]. Thus a brain region might be involved in multiple networks, in which case the time series of that region would be a mixture of signals. In such cases, hard parcellations are unable to adequately address the mixture of signals, since all voxels within a region are assigned the same weight. High-dimensional independent component analysis, on the other hand, takes signal mixtures into account by defining nodes as (potentially overlapping) spatial maps of functionally related voxels [45,46]. Overlapping spatial maps can also be produced using seed-based functional connectivity maps [47] and temporal functional modes [48]. Because spatial maps are often composed of multiple groups of contiguous voxels, and spatial maps often overlap with each other, each network typically describes non-contiguous and overlapping parts of the brain. Depending on the research question, one could represent graph nodes by individual networks. If contiguous regions are desired, each contiguous portion of a network could be described by a separate node. In either case, connectivity can then be estimated between pairs of regions and represented as a graph. The graph could subsequently be analyzed using the methods described in this paper.

Another potential concern related to segmentation is regions with unique internal connectivity patterns. Van den Heuvel and Hulshoff Pol (2010) [47] have shown that voxels in one hemisphere of the motor strip have maximum correlation with voxels in approximately the same location in the contralateral hemisphere. Previous studies have shown that connectivity gradients exist in some parts of the brain [32]. These unique connectivity features are poorly represented by distinct regions that assume uniform intra-region connectivity. It is important to keep this in mind when interpreting the results of connectivity analyses.

The question of segmentation strategy is not trivial and must be considered with great care. Choice of segmentation method and resolution can have a profound influence on network characteristics [49]. It is essential to realize that a poor segmentation can inappropriately group functionally unrelated voxels into the same region, or can split voxels with homogeneous signals into multiple regions.

Whether using a ‘hard parcellation’ or a non-contiguous and overlapping segmentation, if one wishes to analyze multiple subjects simultaneously, it is essential that segmentations correspond across subjects [32,45]. If this basic requirement is met, then the visualizations methods presented in this paper can be applied to graphs produced with the discussed segmentations, provided that a logical grouping of nodes is provided. That being said, it is essential to realize that even though the visualizations may show interesting results, poor segmentations can obscure true connectivity patterns and interpretation of results.

#### Grouping regions of interest

Another issue related to segmentation is how to group regions. Regions can be grouped anatomically [39], which was the strategy used in this study. Regions can also be parcellated using graph-theory based methods. For example, Betzel et al. (2013) [50] used a Markov process to group nodes on multiple scales based on connectivity obtained from diffusion data and evaluated each scaled based on several metrics derived from structural or functional connectivity. In another study, Yeo et al. (2011) [51] used a clustering approach to group nodes using resting-state fMRI data and found node groups that were similar to resting-state networks. Previous studies have shown that functionally-determined networks are highly interpretable and are reproducible across sessions [52] as well as across sites and datasets [53], and as such may provide a reliable parcellation to be used in graph-based brain connectivity studies. The cortical parcellation from Yeo et al. (2011) [51] was used by Betzel et al. (2014) [11] to assess changes in functional an structural connectivity across the lifespan and Sripada et al. (2014) [54] to determine whether attention-deficit/hyperactive disorder causes developmental lag within the connectome as children grow older. In both cases, the borders of ROIs within each group corresponded well with the borders of each of the parcellations. A functionally defined parcellation could be used to group regions from a segmentation that does not have corresponding borders, such as an anatomically-defined segmentation. However, this may not be the best option if one or more ROIs have significant overlap with two networks.

It is important to note that applying an existing cluster-based parcellation to a new dataset assumes that the parcellation is representative of the new data set. It is possible that the composition of node groups changes with age or in different psychiatric states. In the case of task-based fMRI, such as in the MGH data set, it is questionable whether resting-state-network-like groupings are an appropriate choice. To circumvent these issues, study-specific node groups could be computed. However, there is the question of what sample size is required to obtain a reliable estimate of parcellations. Additionally, multiple scales may offer feasible node groups, the use of which likely requires justification in the context of the research question being asked. In spite of the uncertainty that is involved in using data-driven groupings of regions, their functional basis makes them worthy of consideration in future studies assuming that these uncertainties are taken into account when interpreting results.

Whether region groups are decided anatomically, in a data-driven manner, or in another manner, the groupings can easily be used in worm plots and Connectograms to draw conclusions about within- and between-group connectivity. While these figures cannot simultaneously represent node groups of different scales and compositions, they can be used on each scale separately.

#### Multiple testing correction

The methods in this paper used mass univariate testing [12] to look for group differences in brain connectivity on global, lobe-level and individual connection levels. The large amount of tests involved in this approach must be addressed by using some form of multiple testing correction. We addressed this by calculating an “effective number of independent tests” and using that number in a Bonferroni correction [23]. Several similar methods exist [55], along with alternatives such as permutation testing [56].

Even with a liberal method of multiple testing correction, few or no significant tests may be found. While this is largely due to the large number of tests involved, the problem can be magnified by low signal-to-noise ratios in data, which will weaken observed statistical relationships. In these cases, a coarser level of analysis can increase statistical power. For example, the network-based statistic method thresholds individual test statistics of a network, identifies resulting connected components and calculates a p-value for each component. By considering sub-networks rather than individual connections, the network-based statistic gains statistical power, especially when signal-to-noise ratios are low.

Another alternative to mass univariate testing is to use graph theoretical measures [57,58], which can also be used to study group differences and associations with continuous variables, such as age and neurological test scores [11,16,39,59,60]. While the use of graph theoretical measures is a promising area of research, great caution must be exercised when using them to describe group differences or changes relative to a continuous variable. Deficiencies in the construction of input networks, including inappropriate choice of regions defining nodes, might affect graph theoretical measures and their interpretation, and their abstract nature makes it difficult to determine whether observed associations are due to true changes in connectivity or due to confounds [32].

Yet another alternative to mass univariate testing is “seed partial least squares correlation” [61], which combines the ROI correlation matrices of all subjects and performs singular value decomposition to produce new matrices that can provide information about which seed region contributes most to group differences. It is important to note two differences between the correlation matrices from the partial least square method and those used in this study. Firstly, it works with correlations of voxels, and thus requires that all subjects’ brain scans are in the same space, which ignores the individual anatomical and functional differences that exist between subjects [20]. This can be overcome by considering regional time-series instead of voxel time-series, in which case subjects do not need to be in the same space. The second difference is that the partial least squares method considers correlations between two mutually exclusive sets of time-series, and thus correlations between some pairs of time-series are not considered.

The three alternatives to mass univariate testing described here, namely the network-based statistic, graph theory measures and seed partial least squares correlation, are viable alternatives that one might consider when there is a concern about statistical power or signal to noise ratio. They are, however, incapable of identifying individual significant connections. Both univariate testing and methods on a coarser scale are worth considering when feasible and appropriate.

Along with mass univariate testing, the methods presented in this paper might be affected by the effects of multiple testing when considering multiple connectivity metrics. In this study we did not control for multiple comparisons of connectivity metrics because the connectivity metrics that we used are likely highly dependent. That combined with the fact that we only compared four metrics would result in minimal effects of multiple testing correction. However, it is important to realize that not correcting for multiple tests might inflate the false positive rate. Future studies that aim to compare multiple connectivity metrics should evaluate the degree of dependence that exists between metrics and choose an appropriate method of multiple testing correction, especially if the comparisons are used to decide on a metric that best suits their research question.

#### Alternatives to graph-based connectivity

This paper was primarily concerned with connectivity modelled by graphs, where ROIs define nodes and relations between nodes define edges. There are, however, other ways of defining connectivity. For example, functional connectivity is often examined using independent component analysis [62], (fractional) amplitude of low-frequency fluctuation [63] and dual regression [64]. These methods produce spatial maps of voxels with similar dynamics. Spatial maps of FA and MD can also be created from diffusion MRI data. Both measures are used to assess the health of the white matter tracts that are at the core of brain connectivity [65–69]. White et al (2009) [65] discovered white matter “potholes”, which are FA hypo-intensities that are spatially unique to each subject. While pothole locations often do not overlap between subjects, the occurrence of potholes tends to be higher in subject groups than in controls [65,67,68].

The visualizations presented in this paper were not designed to be used with voxel-wise connectivity metrics. The worm plot, however, could be easily used to visualize the results of voxel-wise connectivity studies by representing the statistical test associated with each voxel within a network as a point in the plot. Points can then be grouped by the networks to which they belong. The bi-modal connectivity plots could technically be used with spatial map data. However, to show a bi-modal relationship there should be a one-to-one correspondence between points in the functional and structural maps. Although you can relate corresponding functional and structural voxels to each other, voxel-wise relationships between diffusion and functional maps are rarely of interest in connectivity studies. Rather, the interest lies in the relationship between functionally connected grey matter regions and the white matter tracts that connect them. One could use the bi-modal plots to show relationships between spatial maps originating from the same modality, although this then becomes a uni-modal comparison. For example, one could plot the relationship between z-scores from FA pothole analysis and z-scores of radial diffusivity or mean diffusivity to determine whether a subject group’s FA potholes have corresponding drops in other measures of white matter integrity. Unlike the worm and bi-modal network plots, the connectogram cannot be used with voxel-wise data since it is inherently meant to represent data that is represented as a graph. Of course, one could use the spatial maps from voxel-wise analysis to define regions of interest and subsequently use them to derive graphs, which could then be visualized using all of the presented graphs, but this would then no longer be a voxel-wise analysis.

Whether examining connectivity via graph-based methods or using a voxel-wise approach, an important consideration is whether to use spatial normalization. Spatial normalization requires non-linear registration to a common reference space, thus potentially losing important information related to subjects’ unique anatomical features. The use of spatial normalization assumes spatial overlap of functional activation or white matter abnormalities across subjects. This assumption is often not the case [20,65]. An advantage of graph-based connectivity is that a common set of nodes is the basic requirement to be able to compare subjects. Thus spatial normalization is not a requirement, even though it is sometimes still used. Some voxel-wise methods such as amplitude of low-frequency fluctuation and dual regression are also capable of generating subject specific connectivity maps in subject space. Group voxel-wise comparisons would still need to be carried out in standard space, but the registration can then be done on the subject-specific maps. In this case, connectivity estimation is not affected by normalization. Whether using spatial normalization or not, both spatial map methods and graph-based methods are invaluable tools in the study of brain connectivity.

### Future directions

This paper focused on methods for integrated analysis and visualization of structural and functional connectivity, and provided a proof of concept in rather small pilot studies. In the future we will apply the methodology to larger samples, and will use it to evaluate new methods of defining connectivity.

## Conclusions

A method for integrated analysis and visualization of structural and functional connectivity differences has been proposed. In two pilot studies it could be shown that the approach enabled analysis of group differences, namely between elderly and middle-aged subjects as well as between patients with schizophrenia and controls. The method provides a way to evaluate connectivity metrics against each other for use both uni- and bi-modally.

## Supporting Information

S1 Fig Illustration of figures previously used in connectivity studies. Each sub-figure was generated from data used in this study. Matrix representation (a) can be used to show the contents of a network [11–13]. Each row and column represents an ROI, and each element in the matrix represents a connection between the ROIs in the corresponding row and column. Connectograms (b) show connectivity by arranging ROIs on the outer edge of a circle and representing connections as lines between them [10,14]. 3-dimensional representations (c) represent ROIs as points positioned at their location within the brain, which are connected by lines [7,12,13,15,16]. This visualization requires projection of the 3-dimensional representation into 2-dimensional space. Scatter plots (d) show relationships between structural and functional connectivity [7], where each point represents a connection and in this case color is used to represent groups.
(TIF)

S2 Fig Bar plot of worm plot shifts. One-sample, two-sided t-tests were used to determine the degree to which each worm in the worm plot is shifted away from zero in the (a) RSS and (b) MGH studies for all pairs of region clusters. Groupings include subcortical (Sub), occipital (Occ), parietal (Par), temporal (Temp), frontal (Fro) and the cerebellum (Cb). Cb was not included in the RSS study. On the y-axis is the negative log of the p-value, multiplied by the sign of the corresponding t-test and scaled such that the line indicating p = 0.05 is at the same position for all plots.
(TIF)

S1 Table Regions involved in the proof-of-concept studies, grouped by location in the brain. (DOCX)
