# Supplementary figures and images for: Integrated Analysis and Visualization of Group Differences in Structural and Functional Brain Connectivity: Applications in Typical Ageing and Schizophrenia
Source: PLoS One. 2015 Sep 2;10(9):e0137484. doi: 10.1371/journal.pone.0137484 (PMC4557994; doi:10.1371/journal.pone.0137484)

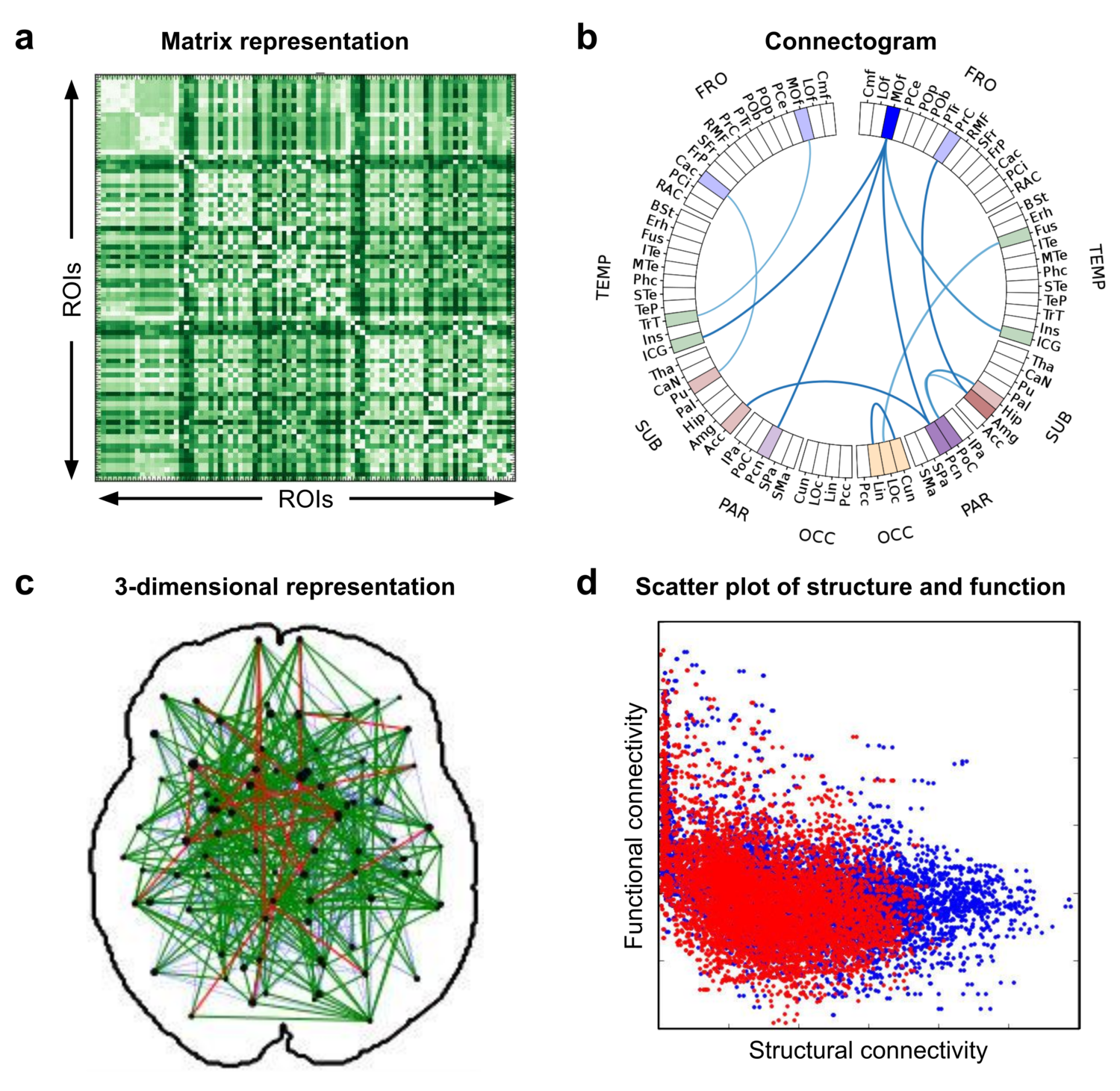

Supplement: S1 Fig — Each sub-figure was generated from data used in this study. Matrix representation (a) can be used to show the contents of a network [11–13]. Each row and column represents an ROI, and each element in the matrix represents a connection between the ROIs in the corresponding row and column. Connectograms (b) show connectivity by arranging ROIs on the outer edge of a circle and representing connections as lines between them [10,14]. 3-dimensional representations (c) represent ROIs as points positioned at their location within the brain, which are connected by lines [7,12,13,15,16]. This visualization requires projection of the 3-dimensional representation into 2-dimensional space. Scatter plots (d) show relationships between structural and functional connectivity [7], where each point represents a connection and in this case color is used to represent groups. (TIF) [file pone.0137484.s001.tif]

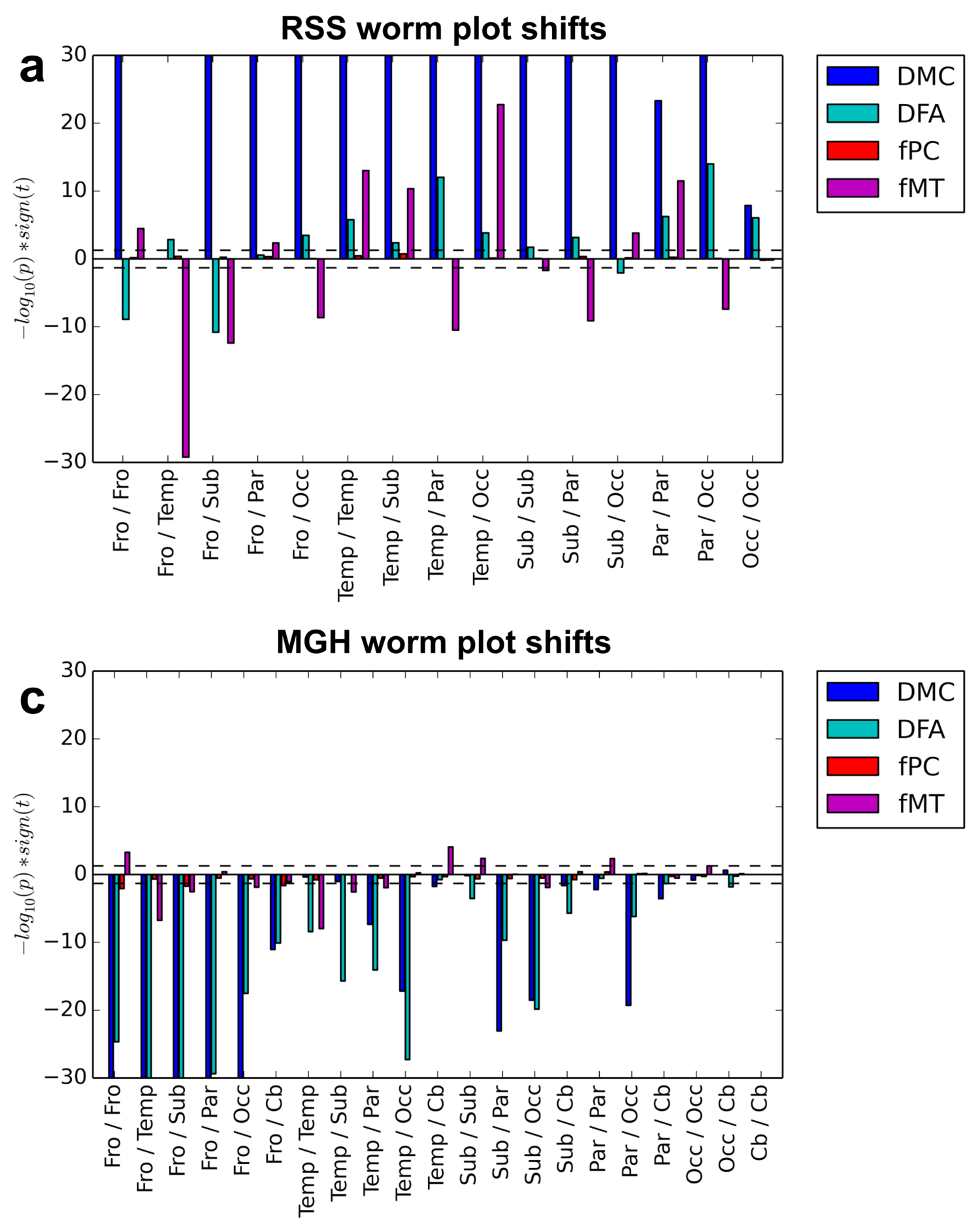

Supplement: S2 Fig — One-sample, two-sided t-tests were used to determine the degree to which each worm in the worm plot is shifted away from zero in the (a) RSS and (b) MGH studies for all pairs of region clusters. Groupings include subcortical (Sub), occipital (Occ), parietal (Par), temporal (Temp), frontal (Fro) and the cerebellum (Cb). Cb was not included in the RSS study. On the y-axis is the negative log of the p-value, multiplied by the sign of the corresponding t-test and scaled such that the line indicating p = 0.05 is at the same position for all plots. (TIF) [file pone.0137484.s002.tif]
