# Supplementary material for: Integrated Analysis and Visualization of Group Differences in Structural and Functional Brain Connectivity: Applications in Typical Ageing and Schizophrenia
Source: PLoS One. 2015 Sep 2;10(9):e0137484. doi: 10.1371/journal.pone.0137484 (PMC4557994; doi:10.1371/journal.pone.0137484)
Supplement: S1 Table — (DOCX) [file pone.0137484.s003.docx]

| **Cluster** | **Region** | **Abbreviation** |
| --- | --- | --- |
| Cerebellum (Cb) * | Cerebellum * | CeB |
| Frontal (Fro) | Caudal anterior cingulate cortex | Cac |
|  | Caudal middle frontal gyrus | Cmf |
|  | Frontal pole | FrP |
|  | Lateral orbitofrontal cortex | LOf |
|  | Medial orbitofrontal cortex | MOf |
|  | Paracentral lobule | PCe |
|  | Pars opercularis | POp |
|  | Pars orbitalis | POb |
|  | Pars triangularis | PTr |
|  | Posterior cingulate gyrus | PCi |
|  | Precentral gyrus | PrC |
|  | Rostral anterior cingulate gyrus | RAC |
|  | Rostral middle frontal gyrus | RMF |
|  | Superior frontal gyrus | SFr |
| Occipital (Occ) | Cuneus | Cun |
|  | Lateral occipital gyrus | LOc |
|  | Lingual gyrus | Lin |
|  | Pericalcarine cortex | Pcc |
| Parietal (Par) | Inferior parietal lobule | IPa |
|  | Postcentral gyrus | PoC |
|  | Precuneus | Pcn |
|  | Superior parietal lobule | SPa |
|  | Supramarginal gyrus | SMa |
| Subcortical (Sub) | Accumbens area | Acc |
|  | Amygdala | Amg |
|  | Caudate | CaN |
|  | Hippocampus | Hip |
|  | Pallidum | Pal |
|  | Putamen | Pu |
|  | Thalamus | Tha |
| Temporal (Temp) | Banks of superior temporal sulcus | BSt |
|  | Entorhinal cortex | Erh |
|  | Fusiform gyrus | Fus |
|  | Inferior temporal gyrus | ITe |
|  | Insula | Ins |
|  | Isthmus of cingulate gyrus | ICG |
|  | Middle temporal gyrus | MTe |
|  | Parahippocampal gyrus | Phc |
|  | Superior temporal gyrus | STe |
|  | Temporal pole | TeP |
|  | Transverse temporal gyrus | TrT |

*Note: the Cerebellum was not included in the RSS study.
